# Dispensing of antibiotics without a prescription by community pharmacies in Nepal: A call for action

**DOI:** 10.1016/j.puhip.2021.100117

**Published:** 2021-04-20

**Authors:** Ramesh Sharma Poudel, Shakti Shrestha, Shital Adhikari

**Affiliations:** Discipline of Pharmacy, Graduate School of Health, University of Technology Sydney, Sydney, NSW, Australia; School of Pharmacy, University of Queensland, Brisbane, QLD, Australia; Department of Internal Medicine, Chitwan Medical College Teaching Hospital, Bharatpur-10, Chitwan, Nepal

Dear Editor,

Increased antimicrobial resistance (AMR) is a global public health concern. AMR is another global pandemic with millions of antibiotic-resistant infections and more than 700,000 deaths each year. If no urgent proactive action is taken, by 2050, antibiotic-resistant infection could cause 10 million deaths and a cumulative loss of 100 trillion USD each year. In Nepal, AMR is a serious public health issue due to the widespread irrational use of antibiotics. Available evidence from Nepal suggests many common pathogenic bacteria are becoming increasingly resistant to most first- and some second-line antibiotics [1]. A WHO report in 2014 stated that in Nepal, 64% *Escherichia* *coli* isolates were resistant to fluoroquinolones, around 38% *Escherichia* *coli* and up to 48% *Klebsiella* *pneumonia* isolates were resistant to third-generation cephalosporins, and prevalence of Methicillin-resistant *Staphylococcus aureus* ranged from 2 to 69% [2]. A 23- year time-trend analysis (1991–2014) of AMR in one of the tertiary care hospitals of Nepal showed there was a significant rise in the proportion of multi-drug resistant (MDR) gram–negative and gram-positive organisms over time [3]. Importantly, this analysis demonstrated there was an annual rise in the proportion of MDR organisms even in patients attending emergency department and outpatient clinic of the study hospital, which indicates the increasing prevalence of MDR organism in the community level. The rise in AMR in Nepal could be attributed to the widespread non-prescription dispensing and use of antibiotics, an insufficient surveillance system for infection controls, and the irrational use of antibiotics in clinical setting, amongst other factors [1].

In Nepal, the physician-patient ratio is approximately 2 per 10,000. Patients mostly bear out-of-pocket expenditures for health service utilization (except in limited circumstances and for specific populations). This may be one of the reasons patients in Nepal often seek medical advice from community pharmacies concerning their general ailments, which could be more common in rural areas. Community pharmacy, by its nature, is an easily accessible healthcare service for the general public. However, there is emerging evidence from several geographical locations of Nepal that community pharmacies have been frequently involved in treating general ailments and self-limiting illnesses with prescription medicines – including antibiotics – without receiving a prescription [[4], [5], [6]]. This would be malpractice. The results of several studies indicate that 67–97% of community pharmacies dispense antibiotics without a prescription [4,[7], [8], [9]]. This also includes the dispensing of multiple antibiotics to a significant proportion of patients [4,7]. Evidence suggest the majority of such pharmacies are either run by non-pharmacy personnel or are not legally registered [[4], [5], [6]], or fail to follow a good pharmacy practice [5] or grossly lack evidence-based databases and related resources that guide the quality and safer use of medications including antibiotics [4,5,7], thus highlighting the shortcomings of pharmacy regulation in Nepal.

Antibiotics commonly dispensed from the community pharmacies without a prescription include the oral dosage forms of cefixime, cepodoxime, amoxicillin, ampicillin ​+ ​cloxacillin, amoxicillin ​+ ​clavulanic acid, azithromycin, ciprofloxacin, ofloxacin, clotrimazole, and metronidazole [4,[6], [7], [8]]. These antibiotics are mainly intended to treat respiratory tract complications (e.g., cough), diarrhoea and dysentery, fevers, skin infections, and urinary tract infections [4,6]. Even injectable antibiotics (e.g. ceftriaxone) were reported to be dispensed without prescriptions [7].

Dispensing of antibiotics without a prescription can significantly increase the likelihood of antibiotics overuse and misuse in minor health ailments and self-limiting illnesses, such as the flu, non-bloody watery diarrhoea, sore throats and colds. Some of the commonly dispensed antibiotics by Nepalese community pharmacies are not even recommended as first-line treatment by the National Antibiotic Treatment Guideline (2014) of Nepal. For instance, cephalosporins are known to be dispensed most commonly without a prescription for enteric fever and urinary tract infections [4,6] but are not recommended by the National Antibiotic Treatment Guideline ​(2014) of Nepal as a first line treatment for such infections [10]. Additionally, community pharmacies frequently dispensed of incomplete courses of antibiotics and many fail to provide information on completing the full course of the therapy [4]. Such overuse, misuse and incomplete use of antibiotic therapy, irrespective of their appropriateness, are the major drivers for increasing emergence of MDR organisms in the community. Data from Nepal also suggests community-acquired infectious diseases such as non-*Salmonella* bacteraemia are developing resistance to a number of antibiotics [3].

This growing AMR and current practice of dispensing of antibiotics without a prescription in Nepal call for urgent action. These may include a strict enforcement of pharmacy regulatory and surveillance systems, particularly those related to the dispensing of antibiotics by community pharmacies, while also consecutively providing education and training related to the emerging AMR and the necessity of supplying antibiotics only with a valid prescription. The Department of Drug Administration, a drug regulatory body in Nepal, should make provisions for heavy fines for such malpractices, enforce record-keeping of dispensed antibiotics, and perform random auditing of these records and obligation of rules by using mystery shoppers. It is equally important that there is increased awareness of the general public on the rational use of antibiotics, and the possible consequences of consuming antibiotics without prescription. Some of the approaches to educating the public may include (but are not limited to) broadcasting information on social and traditional media and using ring tones on cell phones related to the rational use of antibiotics and harm associated with inappropriate use. Utilising local healthcare professionals as watchdogs for reporting non-prescription dispensing of antibiotics by community pharmacies can also be a beneficial means to discourage the malpractices. However, it is important that regulatory authorities take rapid regulatory and correctional action, and where applicable and appropriate, reward whistle-blowers without disclosing their identities. In the long term, this increasing AMR in Nepal also requires a broad approach addressing the social, cultural and behavioural factors that often drive such dispensing practices. Research exploring the factors associated with non-prescription dispensing of antibiotics by community pharmacies and use by the general public is urgently suggested.

## Declaration of interest statement

None.
